# Ultrasound-Guided Superficial Temporal Artery Cannulation for Proximal Arterial Pressure Monitoring During Coarctation Repair With an Aberrant Right Subclavian Artery in a Low-Birth-Weight Neonate

**DOI:** 10.7759/cureus.104532

**Published:** 2026-03-02

**Authors:** Masahiro Wakimoto, Motoi Kumagai, Kenji Suzuki

**Affiliations:** 1 Department of Anesthesiology, Iwate Medical University Hospital, Yahaba, JPN

**Keywords:** aberrant right subclavian artery, aortic cross-clamping, cerebral perfusion monitoring, coarctation of the aorta, complex congenital heart disease, low-birth-weight neonate, neonatal arterial line, subclavian flap aortoplasty, superficial temporal artery, ultrasound-guided cannulation

## Abstract

Coarctation of the aorta (CoA) complex with an aberrant right subclavian artery (ARSA) poses a significant challenge for intraoperative cerebral perfusion assessment because arterial pressure from both the upper and lower extremities becomes unreliable during aortic arch reconstruction. We report a 14-day-old male neonate weighing 1.819 kg with CoA complex, including patent ductus arteriosus (PDA), right-ventricle-type single ventricle, double-outlet right ventricle, single atrium, total anomalous pulmonary venous return (type IIa), mitral atresia, and ARSA, who underwent left subclavian artery flap aortoplasty, PDA clipping, and pulmonary artery banding. Because both subclavian and descending aortic circulations were expected to be unreliable during aortic cross-clamping, the superficial temporal artery (STA) was selected as the only feasible site for real-time cerebral perfusion monitoring. Preoperative ultrasound revealed an extremely small STA (0.47×0.35 mm), yet ultrasound-guided cannulation using a high-frequency linear probe enabled the successful placement of a 24-gauge catheter and provided stable arterial waveforms throughout the procedure. This allowed continuous assessment of cerebral perfusion during aortic cross-clamping and confirmation of the absence of residual pressure gradients after repair. Postoperatively, the patient required diaphragmatic plication for phrenic nerve palsy but otherwise recovered satisfactorily. This case illustrates that ultrasound-guided STA cannulation can be an effective and feasible monitoring option in low-birth-weight infants with complex cardiac anatomy when conventional arterial sites are not usable.

## Introduction

Coarctation of the aorta (CoA) is a congenital narrowing of the aortic isthmus, typically located distal to the left subclavian artery and adjacent to the ductal insertion [1]. CoA frequently occurs as part of a broader spectrum of left-sided obstructive lesions rather than as an isolated anomaly and may coexist with additional congenital cardiac malformations.

Aberrant right subclavian artery (ARSA) is an anatomic variation in which the right subclavian artery arises as the fourth branch of the aortic arch from the proximal descending aorta rather than from the brachiocephalic trunk [2].

The combination of CoA and ARSA creates unique hemodynamic challenges, particularly during aortic arch repair, because arterial pressure obtained from either upper or lower extremities may not accurately reflect true cerebral perfusion. In simple terms, during aortic cross-clamping, the blood pressure measured in the arm or leg may not reflect the pressure supplying the brain; therefore, monitoring a proximal arterial pressure waveform helps clinicians assess whether cerebral perfusion is likely being maintained.

In such circumstances, arterial access through the superficial temporal artery (STA) has been reported as a feasible site for continuous arterial pressure monitoring and as a surrogate for cerebral perfusion, particularly in neonates and infants undergoing complex cardiac surgery [3]. However, despite its potential value, STA cannulation remains uncommon in clinical practice, largely due to technical difficulty, small vessel caliber, and limited published experience, especially in low-birth-weight infants and complex congenital heart disease.

Here, we report a neonatal case of CoA complex with ARSA in which STA cannulation was successfully performed to obtain reliable intraoperative cerebral arterial pressure monitoring. This case highlights the clinical relevance of STA access as an alternative monitoring strategy when conventional arterial sites are unreliable or unavailable.

## Case presentation

A 14-day-old male neonate was referred for the surgical correction of complex congenital heart disease. At birth, his length was 45.2 cm, and his weight was 1.819 kg. He had been followed prenatally for suspected complex cardiac malformation, lateral ventricular enlargement, cleft lip and palate, and intrauterine growth restriction. He was delivered via elective cesarean section at 40 weeks and four days of gestation.

**Figure 1:**
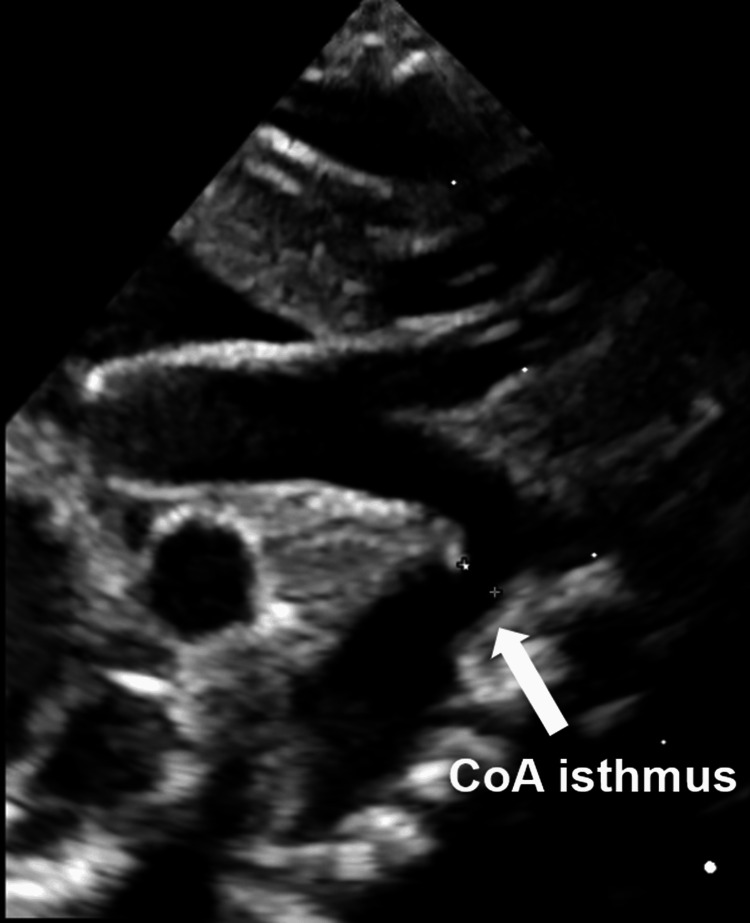
Transthoracic echocardiography demonstrating aortic isthmus narrowing consistent with coarctation Suprasternal notch long-axis view demonstrating discrete narrowing at the aortic isthmus (arrow), consistent with CoA. CoA: coarctation of the aorta

Postnatal echocardiography confirmed the coarctation complex, including patent ductus arteriosus (PDA), single right ventricle, double-outlet right ventricle, single atrium, total anomalous pulmonary venous return (type IIa), and mitral atresia (Figure 1).

**Figure 2:**
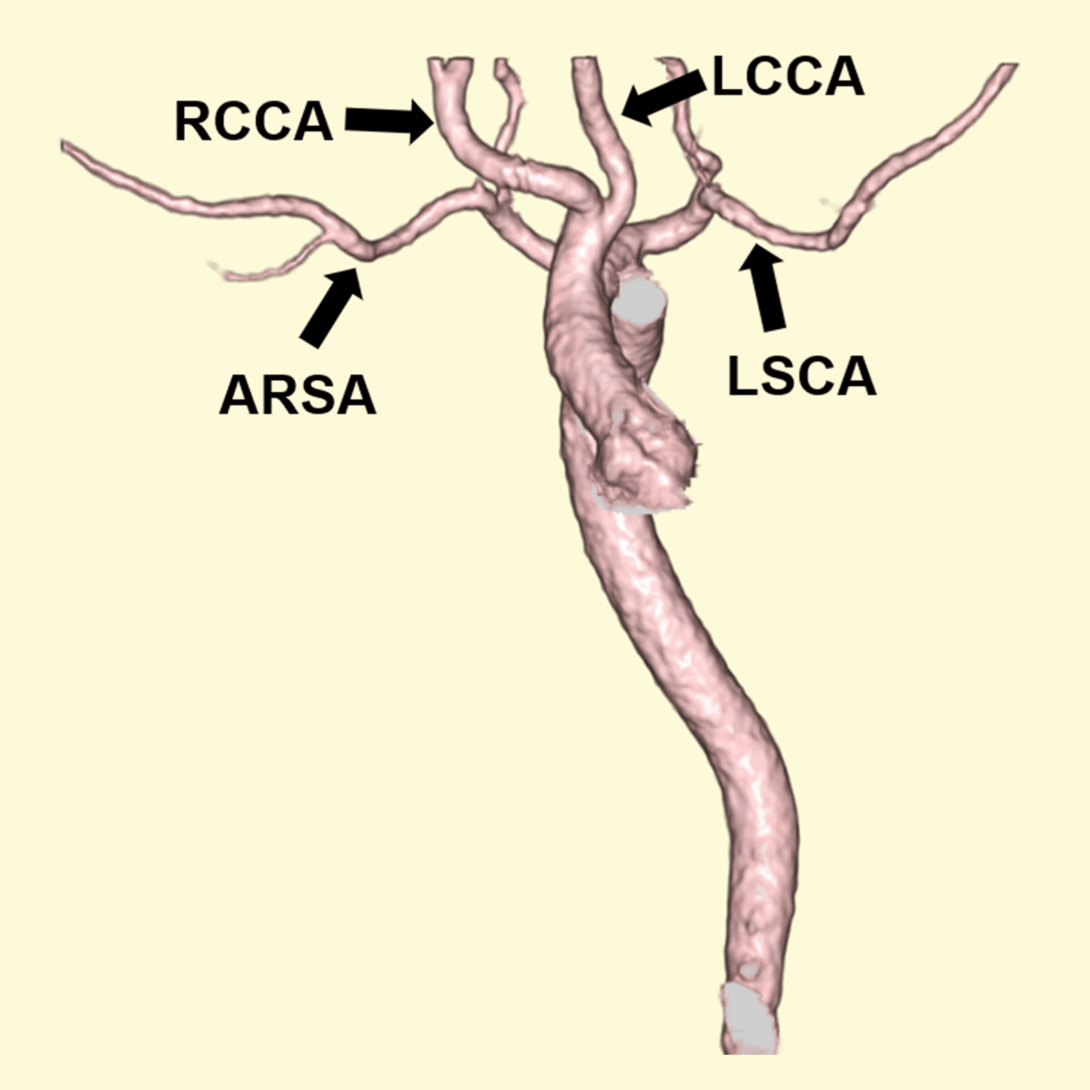
Three-dimensional computed tomography angiography demonstrating aortic arch branching anomaly 3D volume-rendered computed tomography angiography demonstrating the aortic arch branching anomaly with an ARSA. Arrows indicate ARSA, LCCA, LSCA, and RCCA. ARSA: aberrant right subclavian artery; LCCA: left common carotid artery; LSCA: left subclavian artery; RCCA: right common carotid artery

Computed tomography angiography further delineated the aortic arch anatomy, including the aberrant right subclavian artery (Figure 2).

Progressive heart failure prompted surgical intervention consisting of left subclavian artery flap aortoplasty for coarctation repair, ductal clipping, and pulmonary artery banding. Given the planned aortic arch cross-clamping in the setting of ARSA, arterial pressure monitoring from the upper or lower extremities was expected to be unreliable as a surrogate for proximal (cerebral) arterial pressure during key phases of the repair. Therefore, STA cannulation was planned for continuous proximal arterial pressure monitoring.

**Figure 3:**
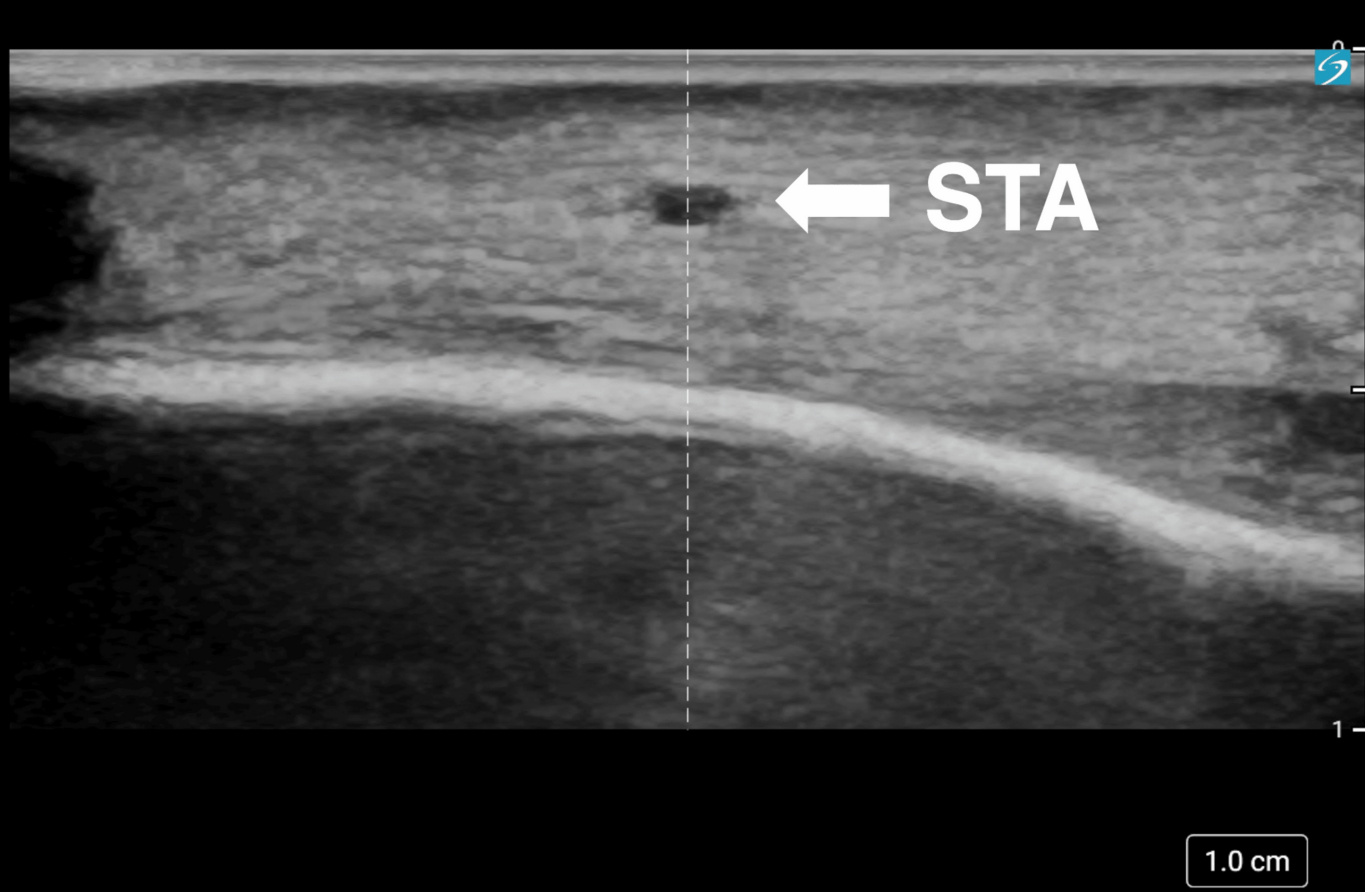
Preoperative ultrasound image of the left STA Short-axis high-frequency ultrasound image of the left STA at a depth of approximately 2 mm from the skin surface. The inner lumen measured 0.47×0.35 mm on preoperative assessment. STA: superficial temporal artery

General anesthesia was induced rapidly with midazolam, fentanyl, and rocuronium, followed by endotracheal intubation. A posterior tibial arterial line, peripheral venous access, urinary catheter, STA line, and central venous catheter via the left internal jugular vein were subsequently secured. Because the patient was positioned in the right lateral decubitus position, the left STA was selected for cannulation. A high-resolution ultrasound system (Sonosite PX, Fujifilm, Tokyo, Japan) with a high-frequency linear probe (L19-5) was used to evaluate the STA. The STA measured 0.47×0.35 mm at a depth of approximately 2 mm from the skin surface on preoperative ultrasound (Figure 3).

Despite the anticipated technical difficulty, a 24-gauge catheter (outer diameter 0.7 mm) designed for peripheral arterial access was successfully inserted under ultrasound guidance, and a stable arterial waveform was obtained.

Surgery was performed through a right lateral thoracotomy at the third intercostal space. The aortic arch and left subclavian artery origin were clamped, and the left subclavian artery was divided to create a flap. The aberrant right subclavian artery, ductus arteriosus, and descending aorta were subsequently cross-clamped. The narrowed aortic segment was incised and repaired using the subclavian flap technique. Pulmonary artery banding was performed using a polytetrafluoroethylene strip.

**Figure 4:**
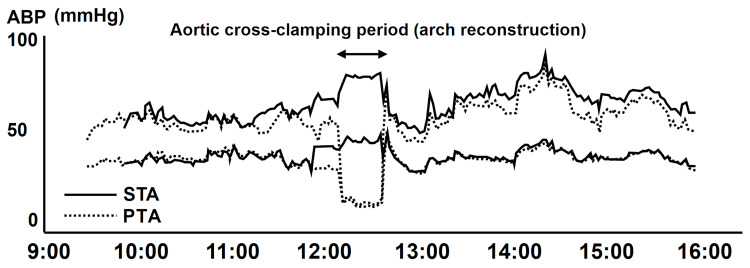
ABP trends during aortic cross-clamping During aortic cross-clamping, the PTA pressure waveform became markedly attenuated, whereas the STA pressure waveform remained continuously available for proximal arterial pressure monitoring. Upper and lower traces represent systolic and diastolic pressures. ABP: arterial blood pressure; PTA: posterior tibial artery; STA: superficial temporal artery

General anesthesia was maintained with midazolam, fentanyl, and rocuronium. The aortic arch cross-clamp time was 45 minutes, during which continuous cerebral perfusion was successfully monitored using the STA pressure waveform. Following coarctation repair, no significant pressure gradient was observed between the upper and lower extremities (Figure 4, Table 1).

**Table 1:** Hemodynamic parameters at key operative time points (STA vs. PTA) STA: superficial temporal artery; PTA: posterior tibial artery; BP: blood pressure; SBP: systolic blood pressure; DBP: diastolic blood pressure; MAP: mean blood pressure; HR: heart rate

Time point	STA BP (mmHg), SBP/DBP (MAP)	PTA BP (mmHg), SBP/DBP (MAP)	HR (beats/min)
Start of surgery	59/39 (48)	58/40 (48)	156
Pre-clamp	72/42 (51)	50/31 (38)	146
During cross-clamp	80/46 (56)	13/12 (13)	150
Post-clamp	61/43 (50)	60/46 (51)	158
End of surgery	73/40 (50)	67/40 (48)	149

Postoperatively, the patient required diaphragmatic plication for phrenic nerve paralysis but otherwise recovered without major complications. He remains under follow-up and is awaiting bidirectional Glenn surgery with Damus-Kaye-Stansel anastomosis, relief of pulmonary venous obstruction, and pulmonary artery reconstruction. Genetic testing revealed partial trisomy of chromosome 12 and partial monosomy of chromosome 17.

## Discussion

CoA occurs in approximately 0.04% of live births and accounts for 6-8% of congenital heart disease, whereas an ARSA, the most common aortic arch branching anomaly, has an estimated prevalence of around 1% in the general population and approximately 3% among patients with congenital heart disease [1,2,4]. In standard CoA repair without anomalous vascular branching, arterial pressure monitoring from the right upper extremity generally reflects cerebral perfusion during aortic cross-clamping. However, when ARSA is present, blood flow to the right upper limb may also be interrupted during surgical repair, rendering both upper- and lower-extremity arterial pressure measurements unreliable. In such cases, direct invasive arterial pressure monitoring via the STA may provide a practical option for approximating proximal (cerebral) arterial pressure and assessing cerebral perfusion [3]. Although STA cannulation has been described as a viable monitoring strategy, it is not without risk. Potential complications include thrombosis, local infection, positive blood cultures, local scarring, and cerebral embolization [3]. Careful patient selection and early removal planning are essential to minimize catheter-related morbidity, especially in neonatal and low-birth-weight populations where vessel fragility is pronounced [5].

In the present case, STA cannulation was anticipated to be technically challenging because the patient was a low-birth-weight neonate (1.819 kg) and the STA lumen measured only 0.47×0.35 mm on preoperative ultrasound imaging. However, recent advances in high-resolution ultrasound technology and increased clinician proficiency in ultrasound-guided arterial access have improved procedural feasibility and safety [6-8]. Ultrasound allowed the precise visualization of the artery, needle trajectory, and catheter placement, contributing to the successful establishment of a stable arterial waveform throughout the procedure. Interestingly, the inner luminal dimensions of the STA (0.47×0.35 mm) appeared smaller than the outer diameter of the 24-gauge catheter (0.7 mm), which, at first glance, suggests an unfavorable catheter-to-artery size relationship. It should be emphasized that ultrasound measurements represent the internal lumen in a single two-dimensional plane and may underestimate the functional vessel caliber, particularly when the artery is partially collapsed or transiently vasoconstricted or the luminal border is difficult to delineate (e.g., due to probe pressure or suboptimal imaging plane). In addition, small muscular arteries in neonates are highly compliant and can accommodate temporary deformation and stretching of the vascular wall during needle entry and catheter placement.

Pediatric data indicate that 24-gauge catheters are commonly used for arterial cannulation in very small arteries, with a high technical success rate when ultrasound guidance is employed, albeit at the cost of a relatively high catheter-to-artery diameter ratio and a theoretical increased risk of occlusion [6-8]. In our case, continuous STA waveforms were maintained without clinical evidence of scalp ischemia. The catheter was removed early postoperatively as a precaution to minimize the risk of catheter-related vascular compromise.

A systematic review and meta-analysis found that ultrasound-guided arterial cannulation improves first-attempt success rates and reduces the number of attempts and complications compared with traditional landmark techniques in children [6]. Similarly, randomized and observational studies in infants have demonstrated higher success rates for ultrasound-guided radial arterial cannulation than for palpation-based techniques [7]. In addition, a recent neonatal case series, including very-low-birth-weight infants, reported high success rates for ultrasound-guided peripheral arterial cannulation without major complications [8].

During aortic cross-clamping, the left posterior tibial arterial pressure waveform became markedly attenuated, whereas the STA waveform remained continuously measurable. Although most prior studies have focused on radial or posterior tibial arteries, our case illustrates that these advantages of high-resolution ultrasound can be extended to extremely small-caliber STA access in low-birth-weight neonates. Bhaskar et al. previously described infants with complex aortic arch pathology and aberrant subclavian anatomy in whom STA cannulation provided reliable proximal arterial pressure monitoring when limb pressures were unreliable during aortic arch reconstruction [3]. Our case adds to this limited body of evidence by demonstrating that STA cannulation can be successfully achieved even in a very small neonate with a body weight of 1.819 kg and an STA diameter of only 0.47×0.35 mm. To our knowledge, reports of STA cannulation in such small-caliber vessels in the context of CoA with ARSA are scarce, and our experience underscores the potential role of STA monitoring in carefully selected high-risk neonatal cardiac cases.

A limitation of this report is the absence of multimodal cerebral monitoring beyond STA pressure. Previous reports suggest that multimodal cerebral monitoring modalities such as near-infrared spectroscopy or transcranial Doppler may enhance the reliability of cerebral perfusion assessment when used in combination with STA pressure monitoring [3,9-10]. In this case, regional cerebral oximetry (O3™ Regional Oximetry, Masimo, Irvine, California, United States) could not be utilized due to limited forehead space. When feasible, such adjunct monitoring may provide additional safety margins in similar high-risk neonatal cardiac surgeries.

## Conclusions

STA cannulation may serve as a valuable and practical arterial monitoring option in neonates with complex congenital heart disease, particularly when anatomical variations, such as an aberrant right subclavian artery, render conventional monitoring sites unreliable. In this case, the use of high-resolution ultrasound enabled successful cannulation despite the extremely small vessel caliber and contributed to safe intraoperative management. This experience suggests that modern ultrasound-guided vascular access techniques may expand the feasibility of STA monitoring in select high-risk neonatal cardiac surgical populations.
